# Complex genetic interaction between glucose sensor HXK1 and E3 SUMO ligase SIZ1 in regulating plant morphogenesis

**DOI:** 10.1080/15592324.2024.2341506

**Published:** 2024-04-12

**Authors:** Sanjay Singh Rawat, Shital Sandhya, Ashverya Laxmi

**Affiliations:** National Institute of Plant Genome Research, New Delhi, India

**Keywords:** Sugar signaling, epistasis, Hexokinase1, post-germination growth arrest, hypocotyl

## Abstract

Sugar signaling forms the basis of metabolic activities crucial for an organism to perform essential life activities. In plants, sugars like glucose, mediate a wide range of physiological responses ranging from seed germination to cell senescence. This has led to the elucidation of cell signaling pathways involving glucose and its counterparts and the mechanism of how these sugars take control over major hormonal pathways such as auxin, ethylene, abscisic acid and cytokinin in Arabidopsis. Plants use HXK1(Hexokinase) as a glucose sensor to modulate changes in photosynthetic gene expression in response to high glucose levels. Other proteins such as SIZ1, a major SUMO E3 ligase have recently been implicated in controlling sugar responses via transcriptional and translational regulation of a wide array of sugar metabolic genes. Here, we show that these two genes work antagonistically and are epistatic in controlling responsiveness toward high glucose conditions.

## Results and discussion

Sugars are key molecules governing major aspects of plant growth and development. Sugar signals interconnect with major phytohormonal networks to induce profound changes in plants physiology throughout their life cycles.^1,2^ In photosynthetic organisms, sugars control these aspects of growth and development through the major sugar sensor, HXK1 that exhibits both catalytic and regulatory/signaling attributes.^3^ However, high exogenous glucose supplementation causes post-germination growth arrest (PGGA), delayed cotyledon expansion and greening of seedlings.^1,4^ Moreover, hypersensitivity to glucose also predisposes the seedlings to ABA, suggesting a link between phytohormonal crosstalk with sugar-mediated signaling.^5^

Earlier, HXK1 mutants named *gin2* (*glucose insensitive* *2*) had been characterized in *Arabidopsis thaliana* as a glucose sensor which functions independently of its metabolism-related activity^1^ and mediates glucose-dependent gene repression of key photosynthetic genes *viz*. chlorophyll *a/b* binding protein (*CAB*), ribulose-1,5-bisphosphate carboxylase (*RBCS*) and carbonic anhydrase (*CAA*).^4^ Furthermore, glucose also regulates expression of other genes independently of HXK1, suggesting the presence of multiple parallel sugar responsive pathways.^4^

Like many other sugar response mutants, the *siz1* (for SAP AND MIZ1 DOMAIN-CONTAINING LIGASE1) mutant shows hypersensitivity toward high exogenous glucose concentrations.^6^ In particular, the *siz1* mutant exhibits enhanced sensitivity toward glucose and sucrose in terms of inhibition of root growth, seed germination and cotyledon greening.^6^ These data point toward the implication of sumoylation of essential proteins at early life stages of a plant in response to sugars since sugars have been implicated in the control of early seed development and seedling establishment.^5^

**Figure 1.**
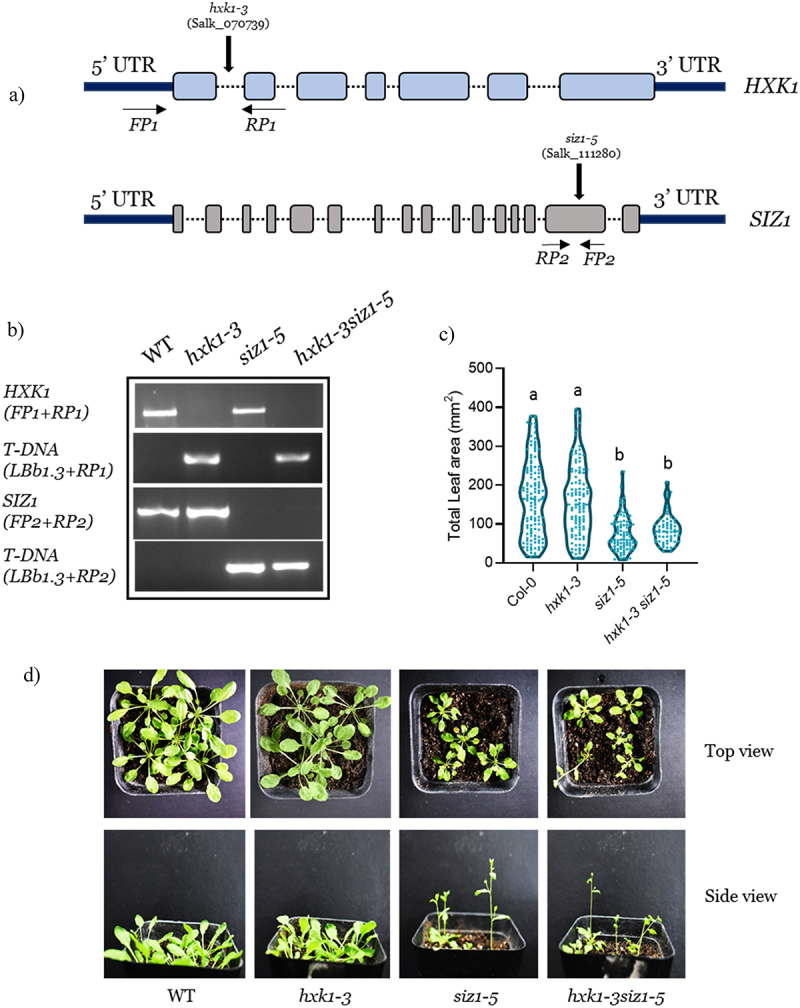
Generation of homozygous double mutant and its phenotypic characterization.

To investigate for the role of HXK1 and SIZ1 in controlling plant growth and development, T-DNA mutants of both *HXK1* (*hxk1–3*, CS69135) and *SIZ1* (*siz1–5*, SALK_111280) (independent mutant allele less characterized than *siz1–2* and *siz1–3*) mutants were obtained from the Arabidopsis Biological Resource Centre (ABRC). Literature mining of these two loss-of-function mutants indicated that for *hxk1–3*, the insertion of T-DNA was in the 1^st^ intron^7^ while for *siz1–5*, 15^th^ exon was disrupted by T-DNA,^8^ in their respective genomes (Figure 1a). The T-DNA insertions in the single mutants and the double mutant was then confirmed by routine genotyping PCR. The results showed that all mutants were homozygous T-DNA insertion lines (Figure 1b). Phenotypic analyses of WT, *hxk1–3*, *siz1–5* and the homozygous double mutant, *hxk1-3siz1–5*, revealed that knockout of either allele or in combination did not affect the normal growth and development of the double mutant (Figure 1c). In particular, *hxk1-3siz1–5* plants flowered early under short days (8 h light/16 h dark) and had rosette and leaf size similar to the *siz1–5* mutants when compared to the WT (Figure 1c,d), suggesting that the *hxk1–3* mutation lies upstream of *siz1–5* mutation.

(a) Schematic illustration of T-DNA insertions in the *hxk1-3* and *siz1-5* alleles (Not drawn to scale). Solid lines represent introns while boxes represent exons. Arrows indicate the location of the primers. (b) Genotyping PCR confirmation of the T-DNA insertions in the *hxk1-3*, *siz1-5* and the double mutant *hxk1-3siz1-5*. Primers used *LBb1.3*-5’ATTTTGCCGATTTCGGAAC3’, *RP1*-5’TCATCAAATGAGGAGGAATCG3’, *RP2*-5’AAAGAGAGAGTGAGCGAAGGG3’. Single mutants were genotyped using the above primers with Forward primers (*HXK1F*-5; TTGTTTTTGATTCCAAATCGG3’ and *SIZ1F*-5’TCCCTCGTAGACATCTGATGG3’). (c) Average leaf area of 35-day-old plants grown under short day conditions (8 h light/16 dark). (d) Growth phenotype of 35-day-old WT, *hxk1-3, siz1-5* and the double mutant *hxk1-3 siz1-5* grown under short days. Different letters indicate statistically significant differences between the means (one-way ANOVA followed by a post hoc Tukey’s honestly significant difference (HSD), *p* < .05).

**Figure 2.**
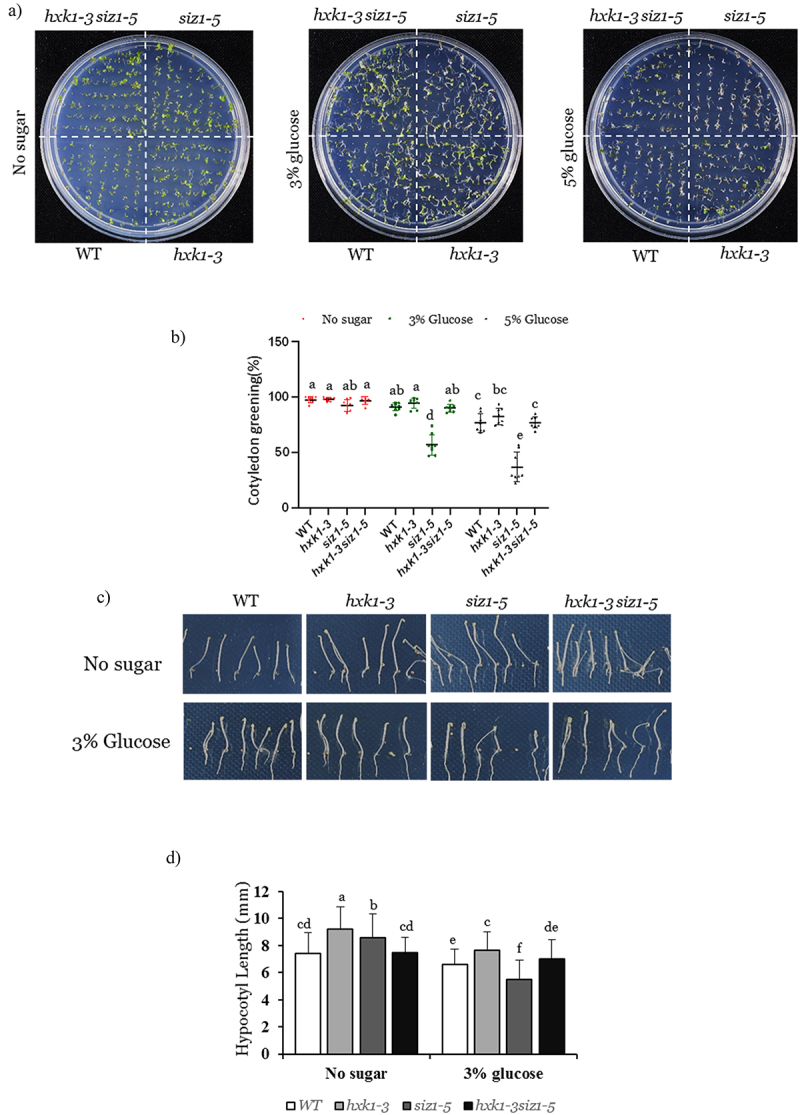
Glucose hypersensitivity of the *siz1–5* mutation is reversed by disruption of *HXK1* signaling.

Since the *siz1* mutation has been shown to be hypersensitive to high exogenous glucose concentrations,^6^ we therefore asked if the disruption of the evolutionary conserved *HXK1* gene in the *siz1* background would confer insensitivity toward glucose. Glucose has been shown to delay seed germination in Arabidopsis,^5^ we therefore first examined percentage seed germination of different genotypes under increasing concentration of glucose. In the absence of glucose (no sugar), there were no significant differences in terms of seed germination between WT (Col-0) and the *hxk1–3*, *siz1–5* and *hxk1–3 siz1–5* mutants (Figure 2). However, at higher glucose concentrations (3% and 5%), PGGA of the *siz1–5* was significantly higher when compared to the other genotypes. In the presence of glucose, the cotyledon greening of the *siz1–5* mutant was also visibly impaired (Figure 2a). Most importantly, this inhibition of seed germination and cotyledon greening of the *siz1–5* was nearly reversed in the *hxk1–3 siz1–5* double mutant (Figure 2a). This suggests that the disruption of the *HXK1* gene in *siz1–5* confers insensitivity toward inhibition of seed germination under high glucose concentrations. Alternatively, the *hxk1* mutation is epistatic to *siz1* mutation in mediating response to inhibition of high glucose concentrations.

(a) Wild-type (WT), *hxk1-3*, *siz1-5* and *hxk1-3siz1-5* seedlings were grown on sugar-free MS medium (no sugar) or with indicated concentrations of glucose. Cotyledon greening percentage was scored after 7 days from the day of seed sowing. Experiments were repeated at least thrice (*n* = 3). (b) Percentage cotyledon greening of the genotypes shown in (a). (c) Representative images of WT, *hxk1-3*, *siz1-5* and *hxk1-3siz1-5* seedlings grown in dark on indicated glucose concentrations. Experiments were repeated at least twice (*n* = 2). (d) Hypocotyl lengths (mm) of the genotypes shown in (d). Different letters indicate statistically significant differences between the means (one-way ANOVA followed by a post hoc Tukey’s honestly significant difference (HSD), *p < .05*).

Next, we studied hypocotyl growth in dark under glucose supplemented conditions of the above mentioned genotypes. As expected, glucose-mediated inhibition of hypocotyl growth inhibition was more in the *siz1–5* mutant than the WT, *hxk1–3* and the double mutant grown in 3% glucose supplemented media (Figure 2cd). Interestingly, the introduction of the *hxk1–3* mutation in the *siz1–5* background resulted in an intermediate phenotype of the *hxk1-3siz1–5* double mutant, suggesting that SIZ1 and H×K1 have distinct and opposite role in sugar sensing and signaling during hypocotyl growth in Arabidopsis (Figure 2c,d).

**Figure 3.**
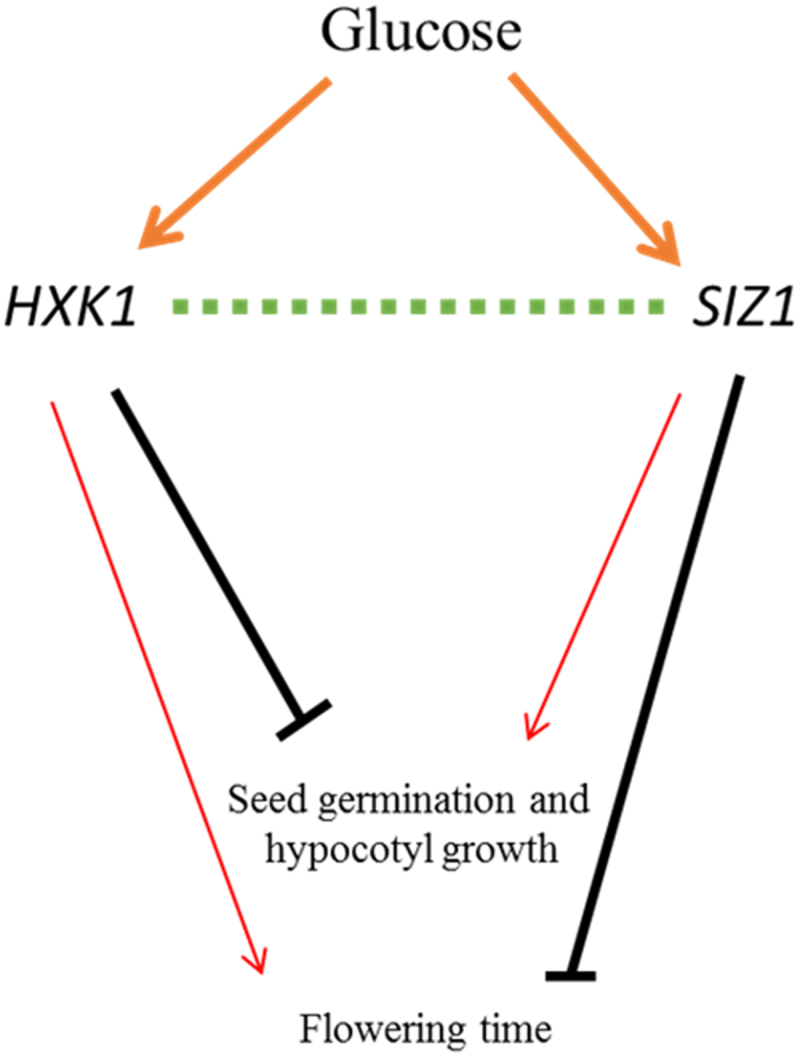
A model for the crosstalk between *HXK1* and *SIZ1* functions.

In conclusion, this report suggests the involvement of HXK1 in SIZ1-modulated sugar signaling is conditionally epistatic across normal overall development (ontogeny) and growth conditions (sugars). Overall, the disruption of HXK1 in *siz1–5* background markedly attenuated the glucose-oversensitive phenotype of the latter in terms of seed germination and hypocotyl elongation under high glucose conditions, suggesting that the function of SIZ1 in sugar signaling might be dependent upon HXK1 activity. Overall, the interaction between these two proteins might govern key developmental aspects of this organism during different phases of the life cycle. In particular, the significance of this report lies in unraveling the molecular machinery behind how plants perceive and react to glucose signals, influencing fundamental processes of seed germination and hypocotyl growth (Figure 3). Moreover, as SIZ1 is involved in salicylic acid signaling, therefore an interconnection between HXK1 and the hormone can be explored.

The glucose sensor HXK1 directly affects normal plant growth and development. Furthermore, SIZ1 is also responsible for plant growth processes, especially cell growth and expansion. Loss-of-function of *SIZ1* confers significantly early flowering and this is also exhibited by the deletion of both *SIZ1* and *HXK1*, suggesting their genetic interaction. Moreover, under conditions of high glucose, HXK1 represses seed germination. Conversely, *siz1* mutation confers the same phenotype, suggesting they might be involved in the same process. However, the current data is insufficient to pinpoint the exact mechanism underscoring their crosstalk. The thickness of the arrows and T-bars indicate the dominance of the particular mutation in the pathway of growth and development below. Dashed lines indicate genetic interaction.

This finding, however, is in contrast to what had been mentioned earlier in the literature.^9^ This discrepancy might have arisen due to the *hxk1* allele being used in Castro et al., 2020,^9^ which differs from the one used in the present study. The *hxk1–3* is a null mutant which has been characterized previously^10^ to be a knockout mutant, the transcript levels being lower than the other mutant alleles, *hxk1–1* and *hxk1–2*.^10,11^ The residual HXK1mRNA expression in the above two lines might be the reason for the obscured genetic analysis.
